# Prevalence of low alkaline phosphatase activity in laboratory assessment: Is hypophosphatasia an underdiagnosed disease?

**DOI:** 10.1186/s13023-021-02084-w

**Published:** 2021-10-28

**Authors:** Tobias Schmidt, Constantin Schmidt, Michael Amling, Jan Kramer, Florian Barvencik

**Affiliations:** 1 Department of Osteology and Biomechanics, University Medical Center Hamburg-Eppendorf, Lottestr. 59, 22529 Hamburg, Germany; 2 LADR Laboratory Group Dr. Kramer and Colleagues, Geesthacht, Germany

**Keywords:** Hypophosphatasia, Alkaline phosphatase, Pyridoxal-5-phosphate, Rare bone disease

## Abstract

**Background:**

Tissue-nonspecific alkaline phosphatase (TNSALP) encoded by the ALPL gene is of particular importance for bone mineralization. Mutation in the ALPL gene can lead to persistent low ALP activity resulting in the rare disease Hypophosphatasia (HPP) that is characterized by disturbed bone and dental mineralization. While severe forms are extremely rare with an estimated prevalence of 1/100.000, recent studies suggest that moderate form caused by heterozygous mutations are much more frequent with an estimated prevalence of 1/508. The purpose of this study was to estimate the prevalence of low AP levels in the population based on laboratory measurements.

**Methods:**

In this study, the prevalence of low AP activity and elevated pyridoxal-5-phosphate (PLP) levels was analyzed in 6.918.126 measurements from 2011 to 2016 at a single laboratory in northern Germany. Only laboratory values of subjects older than 18 years of age were included. Only the first measurement was included, all repeated values were excluded.

**Results:**

In total, 8.46% of the measurements of a total of 6.918.126 values showed a value < 30 U/L. 0.59% of the subjects with an ALP activity below 30 U/L had an additional PLP measurement. Here, 6.09% showed elevated pyridoxal-5-phosphate (PLP) levels. This suggest that 0.52% (1:194) of subjects show laboratory signs of HPP.

**Conclusion:**

These data support the genetic estimation that the prevalence of moderate forms of HPP may be significantly higher than expected. Based on these data, we recommend automatically measurement of PLP in the case of low ALP activity and a notification to the ordering physician that HPP should be included in the differential diagnosis and further exploration is recommended.

## Background

Tissue-nonspecific alkaline phosphatase (TNSALP) encoded by the gene ALPL is widely expressed in different cell types [1]. The enzyme is located on the extracellular surface and dephosphorylates a variety of substrates including inorganic pyrophosphate (PPi) that is a major factor for bone mineralization, and pyridoxal phosphate (PLP) which is required for the synthesis of neurotransmitter like gamma-aminobutyric acid (GABA) [2].

**Fig. 1:**
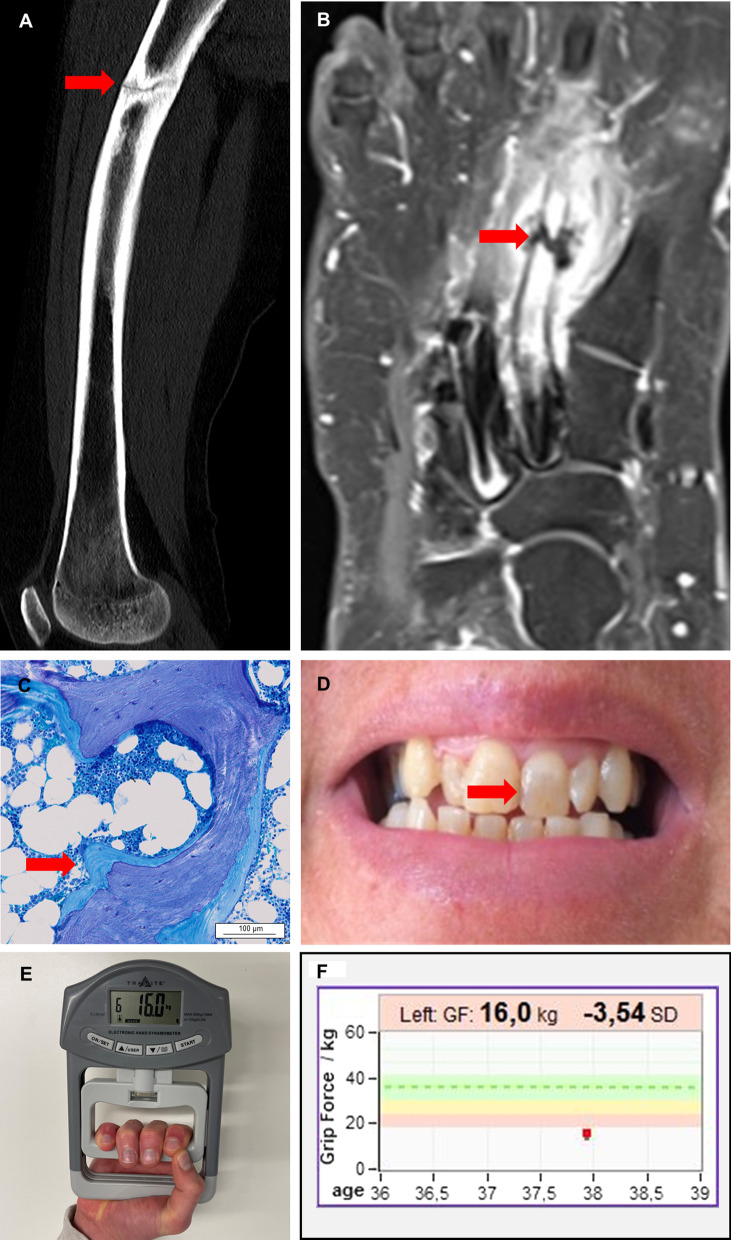
Clinical and histologic characteristics of Hypophosphatasia (HPP). Stress fractures and bone marrow edemas are typical musculoskeletal manifestations of HPP. **a** Stress fracture of the right femur diaphysis. **b** Stress fracture of the right second metatarsale bone. **c** Histological section of a bone biopsy of the os pubis in Toluidine blue staining. Mineralized bone is shown in dark blue. The arrow is showing a thick layer of non-mineralized bone (osteoid) in light blue in the sense of osteomalacia. **d** Typical dental changes with discoloration of teeth due to hypomineralization. **e** and **f** The reduced muscle strength can be objectified using a hand dynamometer. In the example, the patient shows a reduced hand strength (HK) on the left by 3 standard deviations (SD) below the age- and gender-specific reference values

**Fig. 2:**
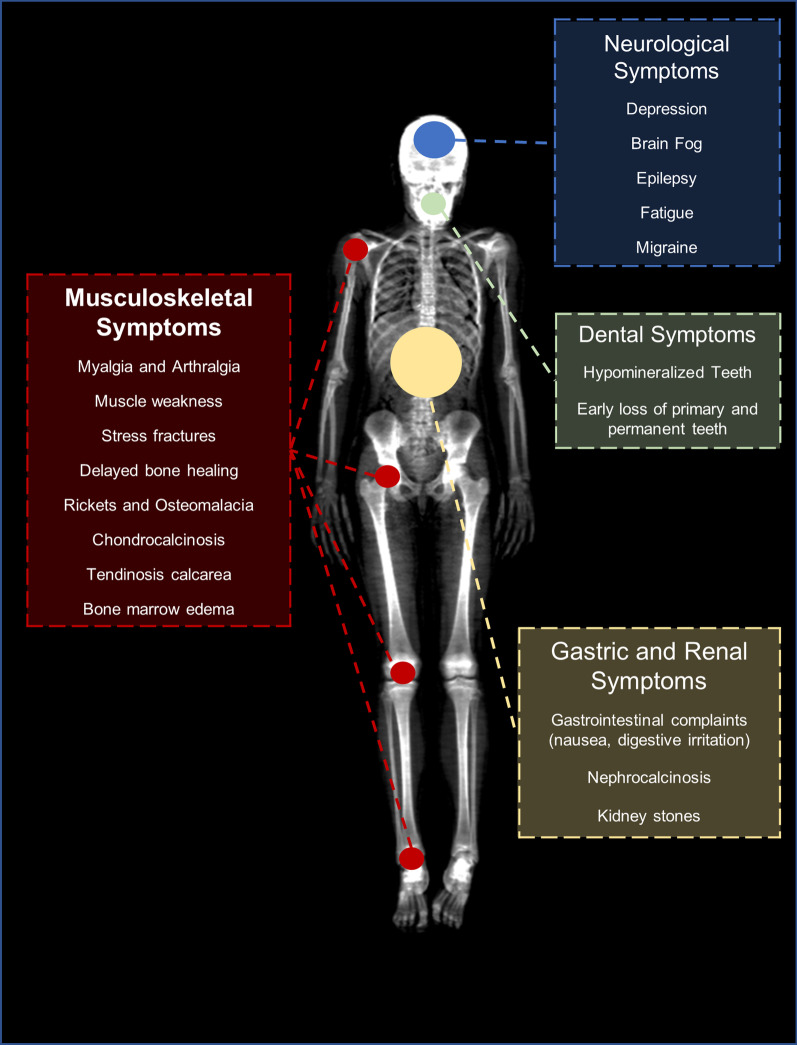
Overview of typical HPP symptoms and their localization. Hypophosphatasia is a metabolic multisystemic disease with various organ systems involved. In addition to musculoskeletal symptoms such as stress fractures, joint and muscle pain, neurological symptoms and gastrointestinal complaints may also occur

Pathogenic mutation within the ALPL gene causes a decrease of ALP activity resulting in the disease Hypophosphatasia (HPP) that causes decreased bone and dental mineralization and is characterized by neurological, musculoskeletal and renal manifestation [3]. The clinical spectrum of HPP is extremely broad ranging from very few clinical symptoms to almost complete absence of bone mineralization in perinatal forms. Figure 1 demonstrates typical symptoms of HPP in adult patients and Fig. 2 shows a graphical summary of possible symptoms. Clinical forms of HPP are classical divided based on age at onset and the existence of bone symptoms and are hereby divided in perinatal, perinatal benign, infantile, childhood, adult and odonto HPP [4]. HPP can strongly affect the life the quality of life in pediatric and adult patients [5, 6]. Recent genetic studies suggest that mild recessive or mild dominant forms of HPP are likely to be much more frequent than previously expected and probably clinical underdiagnosed. Therefore, a recently published study proposes a new classification that takes into account the frequency and severity of the disease. Based on this classification, three forms are proposed; severe HPP, moderate HPP and mild HPP. In this classification the severe form is autosomal recessively inherited, the moderate forms is recessively or dominantly inherited and the mild form dominantly inherited and may reach a frequency of 1/508 in the European population [7, 8]. The wide spectrum of unspecific clinical symptoms in patients with mild forms are genetically caused by different mechanism containing the great variety of the missense mutations, dominant negative effects of missense variants and haploinsufficiency. A total of more than 420 mutations associated with HPP have been described up to date [9]. However, due to the complexity of the genetic background of the disease, it is still difficult to predict the severity of the phenotype with the genetic assessment [7, 10]. Yet, the degree of low ALP activity and the amount of ALP substrate accumulation reflect the severity of HPP [11, 12]. The diagnoses of HPP is confirmed by genetic testing based on the combination of low ALP activity, elevated ALP substrate and the existence of typical clinical symptoms.

While reduced ALP activity is the hallmark of HPP, there are many other reasons for reduced ALP activity such as Osteogenesis imperfecta, postoperative conditions or antiresorptive treatment with bisphosphonates [13]. Table 1 shows a list of different causes for low ALP activity. Therefore, in distinguishing the underlying causes of decreased ALP activity, the determination of ALP substrates plays a crucial role, as the increase clearly indicates HPP. Here, recent studies show that PLP seems to have a much higher sensitivity than other ALP substrates such as phosphoethanolamine (PEA) to diagnose HPP [14] and also correlates with the severity of the disease [12]. This can be explained by the physiological role of PLP. In order to cross plasma membranes PLP must dephosphorylated to pyridoxal which is controlled by ALP. Therefore, elevated plasma levels of PLP in HPP are a result of a failure of extracellular hydrolysis and the measured plasma levels are very sensitive to reduced ALP activity [15].

**Table 1:** Overview of different differential diagnoses for decreased ALP activity. Modified from (13)

Causes of low ALP activity
Hypophosphatasia (HPP)
Osteogenesis imperfecta
Cleidocranial dysplasia
Mseleni joint disease
Vitamin D intoxication
Multiple myeloma
Wilson's disease
Vitamin C deficiency
hypothyroidism
Zn++ or Mg++ deficiency
Cushing's syndrome
Milk-alkali syndrome
Celiac disease
Starvation
Bisphosphonate therapy

In this study we wanted to assess the prevalence of low ALP activity in a large laboratory analysis. We retrospectively screened a large cohort of subjects in Northern Germany and report the number of low ALP activity and elevated PLP values.

## Methods

The laboratory measurements were performed at LADR Centrallab Dr. Kramer & Colleagues, Geesthacht, Germany. Retrospectively all laboratory values of 6.918.126 different subjects older than 18 years were included. The measurements were performed from 2011 to 2016. Repeated values of all subjects were excluded. An AU 5800 clinical chemistry analyzer (Beckman Coulter, Krefeld, Germany), was used to measure serum ALP activity. This instrument measures ALP activity by a kinetic rate method in which a colourless organic phosphate ester substrate (*p*-nitrophenyl phosphate) is hydrolyzed by ALP to the yellow-coloured product *n*-nitrophenol and phosphate at ph 10.4. Changes in absorbance at 410/480 nm are directly proportional to the enzymatic activity of ALP. The normal range for serum ALP in adults is 35–135 IU/L. Pyridoxale-5-Phosphate was meausured by high-performance liquid chromatography following derivatization with fluorometric detection.

Measurements were documented by the laboratory information system MOLIS (Compugroup, Koblenz, Germany) and calculated anonymous by the business intelligence software Deltamaster (Bissantz, Hamburg, Germany) as well as analyzed by the scientific statistical software SigmaPlot (Systat, Erkrath, Germany) and are expressed as total numbers and percentage of measurements. The local ethical committee approved this retrospective study.

## Results

**Fig. 3:**
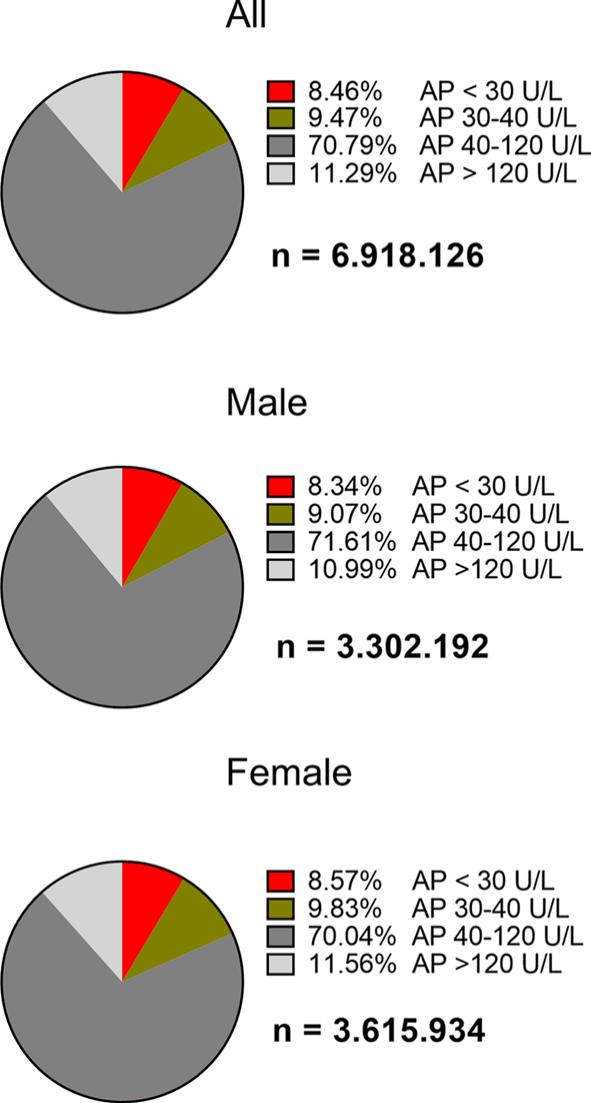
Prevalence of low ALP activity in the general population. Values of ALP activity in 6.918.126 subjects including 3.302.192 male and 3.615.934 female subjects. Percentage contains the first determined ALP value of each subject. All subjects are > 18 years of age

A total of 6.918.126 measurements of different subjects were included in the study. All subjects were above the age of 18. In total, 8.46% showed an AP level below 30 U/L. Another 9.47% of ALP activity measurements were between 30 and 40 U/L (Fig. 3). The percentage of low ALP activity were very similar between men and women (Fig. 3).

**Fig. 4:**
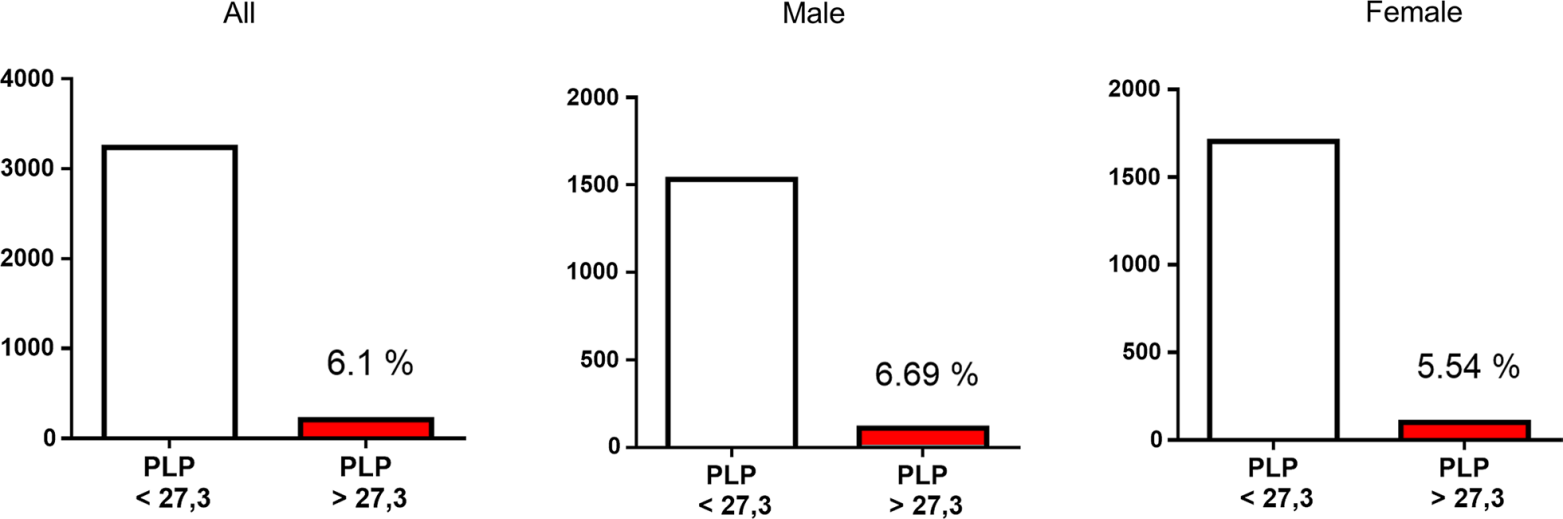
Prevalence of elevated PLP levels in subjects with decreased ALP activity. Values of Pyridoxal-5-phosphate in subjects with ALP activity < 30 U/L. PLP values were available in 0.59% (n = 3.447) of subjects with an ALP activity below 30 < U/L including 0.60% (n = 1.643) of male subjects and 0.58% (n = 1.804) of female subjects

Next to the ALP measurement, we analyzed if available the corresponding PLP values. In total, 0.59% (n = 3.447) of subjects with an ALP activity below 30 < U/L had an additional PLP measurement with 0.60% (n = 1.643) of male subjects and 0.58% (n = 1.804) of female subjects. Among the measurements with low ALP activity, we found 6.09% (n = 210) with elevated PLP levels (Fig. 4). Here the percentage of elevated PLP levels was higher in male subjects than in female subjects (6.69% vs. 5.54%) as shown in Fig. 4 but the difference did not reach significance. Based on the measured number of reduced ALP activity in the cohort and the number of elevated PLP values in this group, we calculated the estimated prevalence of laboratory sings of HPP in the population (Formula: (number of ALP activity < 30 U/L) × (percentage of elevated PLP)/total number of ALP activity measurement). Assuming that the determined values represent the adult population in general it would suggest that 0.52% (1/194) of the population show laboratory signs of HPP. All data with total numbers and percentage are shown in Table 2.

**Table 2:** Presentation of the entire data in total numbers and corresponding percentages

	Total number of ALP activity measurements	Number of ALP activity < 30 U/L	Percentage of ALP activity < 30 U/L (%)	Total number of PLP measurement in the group of ALP activity < 30 U/L	Percentage of measured PLP values in the group of ALP activity < 30 U/L (%)	Total number of elevated PLP in the group of ALP activity < 30 U/L	Percentage of elevated PLP in the group of ALP activity < 30 U/L (%)	Estimated prevalence of ALP activity < 30 U/L and elevated PLP*
All	6.918.126	585.287	8.46	3.447	0.59	210	6.09	0.52% (1/194)
Men	3.302.192	275.402	8.34	1.643	0.60	110	6.69	0.56% (1/179)
Women	3.615.934	309.885	8.57	1.804	0.58	100	5.54	0.48% (1/210)

To calculate the estimated prevalence, the following formula was used * (number of ALP activity < 30 U/L) × (percentage of elevated PLP)/total number of ALP activity measurement

## Discussion

Here we report high frequency of low ALP activity in a large cohort of subjects with laboratory values. It is important to emphasize that these laboratory values were collected in the daily laboratory routine and thus did not serve the purpose of ruling out or diagnose HPP. Similarly, no clear reason can be given for measuring the corresponding PLP values in 0.56% of the subjects with low ALP activity. However, the data show that a considerable proportion of subjects showed reduced ALP activity and correspondingly increased PLP values. This suggest that moderate forms of HPP with borderline ALP activity and increased ALP substrates might be much more frequent than expected and maybe even higher than genetically estimated [16].

In clinical routine, lowered ALP values are often overlooked, as most pathological conditions and diseases such as osteomalacia or cholestasis are associated with elevated values and many laboratories do not provide lower reference values. This is a serious clinical problem that makes the diagnosis of HPP difficult and often leads to pronounced delays in therapy. However, it is important to stress that low ALP values are not synonymous with HPP. Other clinical conditions such as Cleidocranial dysplasia, Mseleni joint disease, Osteogenesis imperfecta, Cushing’s disease, Wilson’s disease, postoperative conditions or medical treatment such as antiresorptive treatment with bisphosphonate or denosumab are also associated with low ALP activity [13]. Table 1 demonstrates a list of conditions associated with low ALP levels. However, a recent study reported that half of the adult patients presenting with unexplained low ALP levels had a mutation in the ALPL gene [14]. Yet, the combination of low ALP activity and elevated PLP levels is highly suspicious for HPP even though vitamin B6 supplementation could not be excluded. In our data 6.1% (n = 210) of subjects with a low ALP activity had elevated PLP levels. This suggest that 0.52% (1/194) of the population shows laboratory signs of HPP.

In a recent study, pathogenic ALPL mutations were comprehensively analyzed by using 3D models and functional test. Here, mild HPP without dominant negative effects were identified as a new clinical entity with a high prevalence in the European population and estimated at 1/254 with a penetrance of 50% resulting in a prevalence of 1/508 [8]. This is caused by different genetic mechanism including dominant negative effects, interaction with collagen and haploinsufficiency [8]. In this study, we support these genetic conclusions by providing evidence from a laboratory analysis suggesting that mild adult HPP has a significantly higher prevalence than expected [16].

Our study has many limitations due to the study design. A major limitation of this study is that we only report the first ALP measurement of each subject and not the rate of persistently lowered ALP activity. Therefore, no statement can be made if some low ALP values were only temporarily reduced, e.g. in bisphosphonate therapy, or whether a permanent reduction is present. Hence, some of the subjects with low ALP activity might be above the lower cut off level in a second measurement. In this context it is important mention that the diagnoses of HPP always requires measurement of persistently low ALP activity. This could be one reason, that our finding that 8.46% of the laboratory cohort fell below the lower range of normal for adults is significant higher than other reports [17]. A recent study reported persistently low levels in 0.12% in the Spanish Population [18] which is a considerable higher prevalence than earlier estimation of the prevalence. Nevertheless, as stated above recent genetic data support the hypothesis that mild forms of HPP are more frequent than earlier expected.

Another limitation of our study are the missing clinical information. It would be very interesting to analyze if clinical symptoms of subjects with low ALP activity are present which would allow to assess the real prevalence of mild HPP in the general population. Similarly, it is not possible to judge whether the measurement of PLP values is related to the ALP measurement in the case of clinical suspicion of HPP or because of other reasons.

In this study, we intentionally included only patients over the age of 18. One reason for this is the difficulty in defining cut-off values in children and adolescents due to the temporary increase in bone metabolism at different ages [19]. Nevertheless, it is very important to point out that a diagnosis in childhood and adolescence is of crucial importance in order to initiate a therapy in time and to prevent severe consequences. It is of highest importance that when the values are measured, the associated reference values at the different ages are provided.

Nonetheless all limitations, our data emphasize that a significant number of people show borderline or low ALP activity and correspondingly increased PLP values. The diagnosis of HPP requires persistently decreased ALP activity, increased ALP substrates and at least one typically symptom of HPP such as insufficiency fractures, bone healing complication, muscle pain or dental complication and most suitable genetic confirmation of an ALPL mutation [3]. In mild forms of HPP these symptoms can be very unspecific such as low muscle strength or muscle pain and are often misdiagnosed as fibromyalgia or Polymyalgia rheumatica [20]. Therefore, it is quite conceivable that an underestimated number of patients with these symptoms suffer from a clinical mild form of HPP.

## Conclusion

Based on these data and recent reports on a higher prevalence of mild forms of HPP we would recommend a special procedure for laboratory testing of ALP activity; if low ALP activity is measured in the laboratory, PLP testing should be offered if suitable material samples are available in the laboratory with the same order. Otherwise, low ALP levels should be commented on the need for PLP testing. In the case of an increase of PLP, an information should then be given to the ordering physician that HPP is suspected and further diagnosis is recommended. This procedure could also be used to make an exact analysis of the prevalence of mild forms of HPP in the future.

## Data Availability

The datasets used and/or analysed during the current study are available from the corresponding author on reasonable request.
